# The History and Capabilities of Herbal Simples

**Published:** 1891-01-17

**Authors:** W. T. Fernie


### The History and Capabilities of Herbal Simples.

By W. T. Fernie, M.D.

### XXVII.—THE COMMON DAISY.

Our English daisy is a composite flower, which is called in the glossaries "gowan." This means in the northern counties, a yellow flower, common in moist meadows, and is also applied to buttercups, hawkweeds, and dandelions. Botanically the daisy plant is named "Bellis perennis," perhaps from the Latin word, *bellus*, handsome. "A silver shield with boss of gold," "the constellated flower that never sets." Or, more probably from "bellis," in fields of battle, because of its fame to heal the wounds of soldiers. Perennis implies that though the rose has but a summer reign, the daisy never dies. This flower is also called Bainwort, because beloved by children, and Consound by reason of its consolidating and vulnerary virtues. The whole plant has been carefully and exhaustively proved for medicinal purposes by Dr. Thomas, of Chester; and a remedial tincture is now made from it by some of our leading druggists. Gerarde says, "daisies do mitigate all kinds of pain, especially in the joints, and gout proceeding from a hot humor, if stamped with new butter, and applied upon the pained place." And "the leaves of daisies used among potherbs do make the belly soluble." Pliny tells us the daisy was used in his time, with mugwort, as a resolvent to scrofulous tumours. In mediæval times this flower was worn at a tournament by a knight in his scarf, it being reckoned an emblem of fidelity. The leaves are acrid and pungent, being ungrateful to cattle, and even rejected by geese. These, and the flowers when chewed experimentally, have provoked giddiness and pains in the arms, as if from coming boils; also a development of boils, "dark, fiery, and very sore," on the back of the neck, and outside the jaws. For preventing or aborting the same distressing symptoms when they begin to occur spontaneously, the tincture of daisies should be given in doses of ten drops three times a day in water. The flowers and leaves are found to afford a considerable quantity of oil, and of ammoniacal salts. The root was named "consolida minima" by the older physicians. Fabricius speaks of its efficacy in curing wounds and contusions. A decoction of the leaves and flowers was given internally; and the bruised herb with lard was applied outside. "The leaves stamped do take away bruises and swellings, whereupon it was called in old time Bruisewort." If eaten as a spring salad, or boiled like spinach, the leaves are pungent, and slightly laxative. Being a diminutive plant, with roots to correspond, the daisy, on the doctrine of signatures, was formerly thought to arrest the bodily growth, if taken for this purpose. Therefore its roots boiled in broth were given to young puppies, so as to keep them of a small size. For the same reason the fairy Milkah fed her royal foster-child on this plant, "that his height might not exceed that of a pigmy."

"She robbed dwarf elders of their fragrant fruit,
And fed him early with the daisy-root,
Whence through his veins the powerful juices ran,
And formed the beauteous miniature of man."

Daisies were also said of old to be under the dominion of Venus, and to be, therefore, good in curing all the pains caused by that fair goddess, particularly those of the breast. A quaint author writes: "For this reason we recommend all *lackadaisical* swains to hasten to the meadows, and there to thank nature devoutly for having scattered this plant with so bountiful a hand." The modest daisy closes its petals at night, and folds them over the yellow disc in rainy weather. A saying goes that it is not spring till you can put your foot on twelve of these flowers. In the new world of America the place of our daisy is taken by that of its more developed congener, the Michaelmas daisy, with brilliantly coloured rays of lilac and mauve. But give me none the less our hardy little native, with its "eyes of silver everywhere disclosed, and with pupils of molten gold, surmounting an emerald stalk." It is curious there is no Greek word for the daisy, and the homely flower fails to spangle the fields in the south-east of Europe. Together with Leigh Hunt I "always like to notice what the Greeks said to things; because they had a sentiment in each of their pleasures." In India our common daisy is carefully cherished in a garden pot. Another name borne by the daisy is Herb Margaret. This is because it used to be employed for the illnesses of females, who came specially under the care of St. Margaret of Cortona. For a like reason the moon daisy has been named Maudlin-wort, from St. Mary Magdalene; and as resembling the full moon. This luminary is identified with the Egyptian goddess Artemis, who particularly governed the female health; an office assigned to Saint Margaret in Roman Catholic mythology. Lady Margaret, Countess of Richmond, bore as her quartering, three white daisies on a green turf. Chaucer was loud in his praises of the daisy, as "ever ilike faire, and fresh of hewe"; "the eye of day"; "the Empresse, and the floure of floures all." In his beautiful poem of the "Flower and the Leaf" he thus pursues the same theme—

"And at the laste there began anon
A lady for to sing quite womanly,
A bargaret in praising the daisie;
For, as methought, among her notës sweet,
She said 'Si douset est la Margarete.'"

Structurally the simple daisy ranks supreme in the first line of plant economy. It is of a higher type than the tall pine, or the spreading oak tree. Consisting at first of many minute yellow flowers, insignificantly small, it has banded these close together on a common stalk, so as to acquire the importance of size. Next it has changed its outer ring of yellow florets to white, (at the sacrifice of their reproductive organs), the better to attract visits from fertilising insects to the flower at large; and even bidding for still higher colouring by gaining pink tips to its outer white florets. Lastly, each central yellow floret has advanced in form from a flat strip to a tubular shape, fitted only for the higher class of insects. Now, therefore, the daisy stands biologically in relation to other plants as man does towards other animals. It has bravely made its way over nearly all the world; and in the vegetable kingdom its enterprising tribe is the dominant "cock of the walk." Taken altogether, this staunch little herb, for its loyal family instincts, its indomitable courage, and its healing virtues on the field of battle, well merits such a distinction as that conferred by Scott on his hero in Marmion—

"So faithful in love, so dauntless in war,
'Tis a Knight among plants, like the young Lochinvar."

